# Effect of a synbiotic on the response to seasonal influenza vaccination is strongly influenced by degree of immunosenescence

**DOI:** 10.1186/s12979-016-0061-4

**Published:** 2016-03-15

**Authors:** Agnieszka Przemska-Kosicka, Caroline E. Childs, Sumia Enani, Catherine Maidens, Honglin Dong, Iman Bin Dayel, Kieran Tuohy, Susan Todd, Margot A. Gosney, Parveen Yaqoob

**Affiliations:** Department of Food and Nutritional Sciences, University of Reading, PO Box 226, Whiteknights, Reading, Berkshire RG6 6AP UK; Department of Mathematics and Statistics, University of Reading, Reading, RG6 6AP UK; Department of Food Quality and Nutrition, Research and Innovation Centre, Fondazione Edmund Mach, via E. Mach, 1, San Michele all’Adige, Trento, 38010 Italy

**Keywords:** Ageing, Influenza, Prebiotic, Probiotic, Vaccination

## Abstract

**Background:**

Ageing increases risk of respiratory infections and impairs the response to influenza vaccination. Pre- and probiotics offer an opportunity to modulate anti-viral defenses and the response to vaccination via alteration of the gut microbiota. This study investigated the effect of a novel probiotic, *Bifidobacterium longum bv. infantis* CCUG 52,486, combined with a prebiotic, gluco-oligosaccharide (*B. longum* + Gl-OS), on the response to seasonal influenza vaccination in young and older subjects in a double-blind, randomized controlled trial, taking into account the influence of immunosenescence markers at baseline.

**Results:**

Vaccination resulted in a significant increase in total antibody titres, vaccine-specific IgA, IgM and IgG and seroprotection to all three subunits of the vaccine in both young and older subjects, and in general, the increases in young subjects were greater. There was little effect of the synbiotic, although it tended to reduce seroconversion to the Brisbane subunit of the vaccine and the vaccine-specific IgG response in older subjects. Immunological characterization revealed that older subjects randomized to the synbiotic had a significantly higher number of senescent (CD28^−^CD57^+^) helper T cells at baseline compared with those randomized to the placebo, and they also had significantly higher plasma levels of anti-CMV IgG and a greater tendency for CMV seropositivity. Moreover, higher numbers of CD28^−^CD57^+^ helper T cells were associated with failure to seroconvert to Brisbane, strongly suggesting that the subjects randomized to the synbiotic were already at a significant disadvantage in terms of likely ability to respond to the vaccine compared with those randomized to the placebo.

**Conclusions:**

Ageing was associated with marked impairment of the antibody response to influenza vaccination in older subjects and the synbiotic failed to reverse this impairment. However, the older subjects randomized to the synbiotic were at a significant disadvantage due to a greater degree of immunosenscence at baseline compared with those randomized to the placebo. Thus, baseline differences in immunosenescence between the randomized groups are likely to have influenced the outcome of the intervention, highlighting the need for detailed immunological characterization of subjects prior to interventions.

**Trial registration:**

Clinicaltrials.gov NCT01066377.

**Electronic supplementary material:**

The online version of this article (doi:10.1186/s12979-016-0061-4) contains supplementary material, which is available to authorized users.

## Background

Influenza is a major cause of death in older people and while vaccination offers a prophylactic solution for preventing infection and associated complications, immunosenescence significantly impairs vaccine efficacy [1]. Potential adjuvants and dietary strategies to improve the immune response to influenza vaccines are therefore of interest, particularly in older people. Emerging evidence suggests that the resident gut microbiota plays an influential role in shaping antiviral defenses and modulating the outcome of viral infections [2], and this forms the basis for the hypothesis that pre- and probiotics may modulate responses to infection or vaccination.

Trials investigating the use of probiotics in prevention of common respiratory illnesses have produced mixed results [3], although a recent systematic review concluded that they significantly reduce episodes of acute URTI and antibiotic usage in infants and young to middle-aged adults [4]. Response to vaccination is increasingly being used as a surrogate for the response to infection [5]. The majority of studies investigating the impact of probiotics on responses to vaccination have been conducted in healthy adults, and some show borderline effects of probiotics on serum or salivary IgA titres, although the clinical relevance is not clear [6]. Studies in infants and in elderly subjects, particularly those examining the response to influenza vaccination, are very limited, as are studies on the effects of prebiotics on immune function [7] and vaccination [6]. Since ageing is associated with reduced biodiversity and compromised stability of the gut microbiota [8], as well as immunosenescence, older individuals may derive particular benefit from intervention with pre- and/or probiotics.

In selecting probiotics specifically for older individuals, Dominguez-Bello et al. [9] suggest that “the healthy old, rather than the healthy young, are the best donors of probiotic species for old individuals”, because they may be better suited to colonization and establishment of a new equilibrium within an aged microbial community. The strain *Bifidobacterium longum bv. infantis* CCUG 52,486 fits this brief because it was originally isolated from a cohort of very healthy elderly subjects (independent life-style, free of chronic disease, and aged 90 years or over) in Italy as part of the CROWNALIFE EU FP5 project [10], it has been demonstrated to have particular ecological fitness and anti-pathogenic effects in vitro [11], and it has immunomodulatory effects which are strongly influenced by the age of the host [12]. Furthermore, this strain has been fully genome sequenced so that genetic traits can potentially be related to biological effects. In this study, we examine the ability of *Bifidobacterium longum bv. infantis* CCUG 52,486, combined with a gluco-oligosaccharide prebiotic (Gl-OS), to modulate the response to seasonal influenza vaccination in a cohort of healthy young and older subjects. We also examine the extent to which baseline markers of immunosenescence influenced the outcome of the intervention.

## Results

### Subject characteristics

**Fig. 1:**
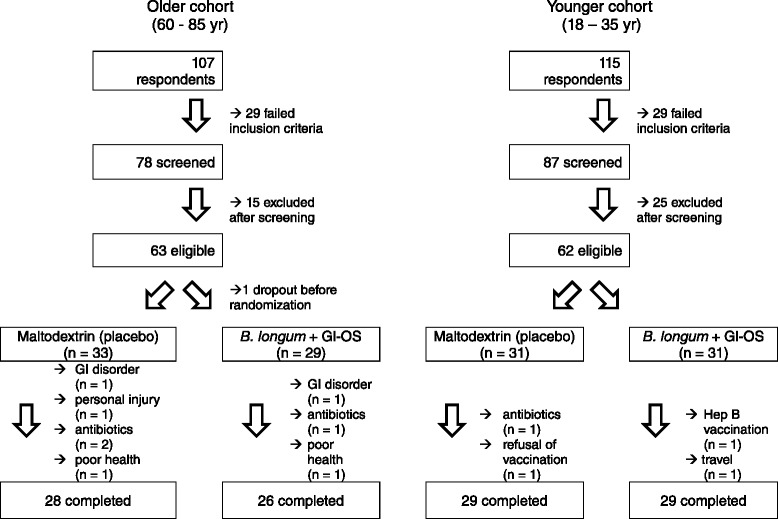
Recruitment flow diagram

The characteristics of the subjects recruited to the study are described in Table 1. Of the 125 volunteers who started the trial, 112 completed (Fig. 1). There were no differences in baseline characteristics between treatment groups within the young or older cohorts (data not shown).

**Table 1:** Baseline characteristics of subjects recruited to a double-blind, placebo-controlled, randomised study of *Bifidobacterium longum bv. infantis* CCUG 52,486 combined with gluco-oligosaccharide

	Older cohort	Younger cohort
	(*n* = 63)	(*n* = 62)
Gender	18♂45♀	23♂39♀
Smoker	4/63	13/62
	*mean (SD)*	
Age	69 (5)	26 (4)
BMI (kg/m^2^)	27 (3)	23 (3)
Waist (cm)	94 (16)	80 (9)
Weight (kg)	74 (13)	68 (13)
Height (m)	1.7 (0.1)	1.7 (0.1)
BP systolic (mmHg)	138 (17)	122 (11)
BP diastolic (mmHg)	78 (9)	74 (8)
Pulse (bpm)	70 (11)	76 (10)

### Effect of *B. longum* + Gl-OS on vaccine subunit-specific total antibody responses to seasonal influenza vaccination

#### Total antibody response to the H1N1 strain

**Fig. 2:**
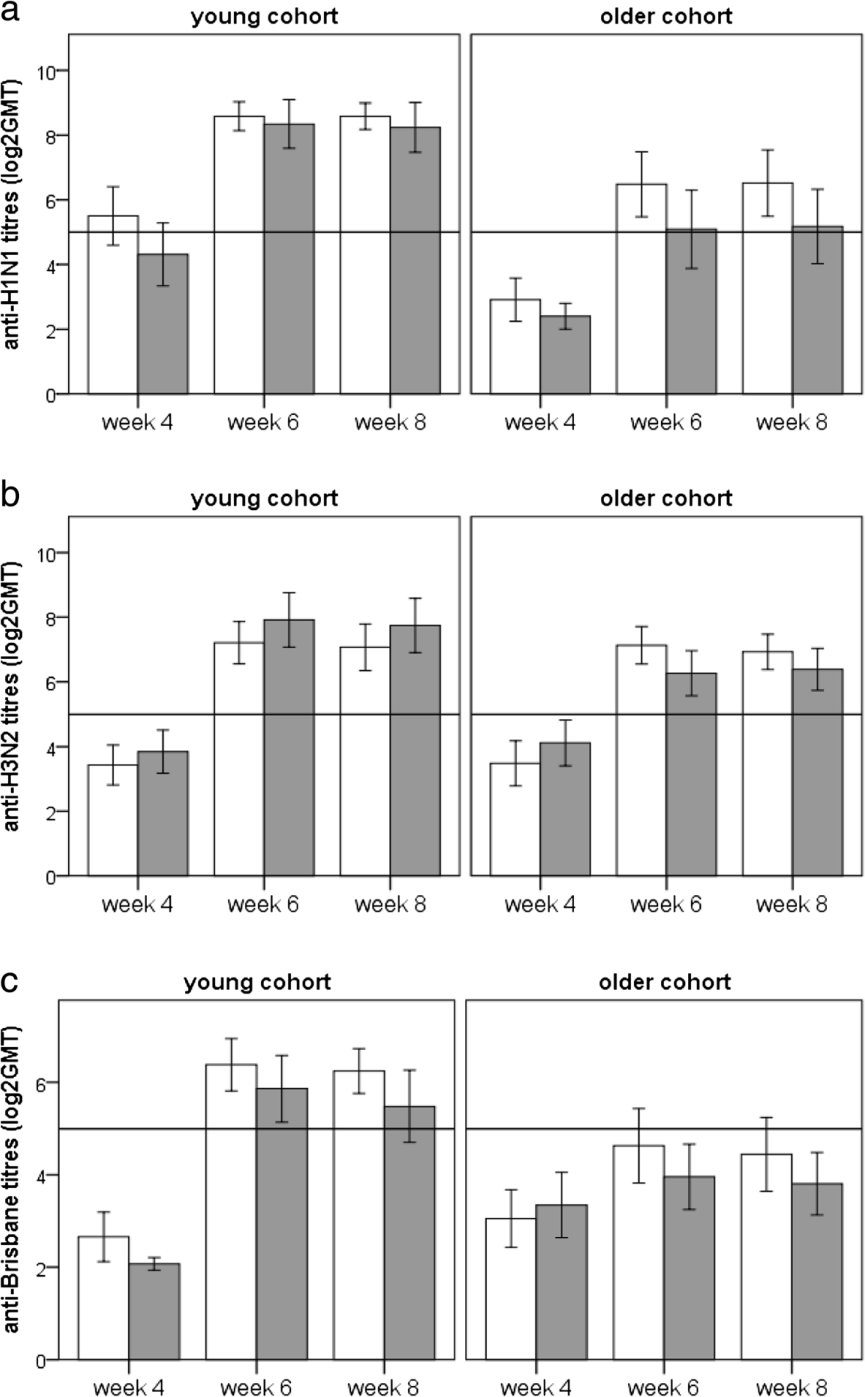
Effect of *B. longum* + GI-OS on antibody responses to the influenza vaccine in young and older subjects. Data are log_2_ transformed geometric mean antibody titres (GMT) (±2SEM) at baseline and weeks 6 and 8 for *n* = 54–58 subjects per group. □ Maltodextrin,  *B. longum* + Gl-OS. The reference line at the Y axis indicates seroprotection (GMT = 32). Data were analysed using a Linear Mixed Model (LMM) with fixed factors of time, age and treatment. **a** H1N1; For the LMM, there were significant effects of age (*p* < 0.001) and time (*p* < 0.001) and a trend for a treatment effect (*p* < 0.02). For data split by cohorts, there was a significant effect of time in both cohorts (*p* < 0.001), but no significant effect of treatment. **b** H3N2; for the LMM overall, there was a significant effect of time (*p* < 0.001) and an age*time interaction (*p* < 0.01). For data split by cohort there was a significant effect of time (*p* < 0.001) and a time*treatment interaction (*p* < 0.01) in the older cohort, and a significant effect of time (*p* < 0.001) in the young cohort. **c** Brisbane; for the LMM overall, there were significant effects of age (*p* < 0.005), time (*p* < 0.001) and an age*time interaction (*p* < 0.001). For data split by cohort, there was a significant effect of time (*p* < 0.001) and a time*treatment interaction (*p* < 0.01) in the older cohort, and a significant effect of time (*p* < 0.001) in the young cohort. There was a borderline effect of treatment (*p* < 0.05)

Vaccination resulted in a significant increase in antibodies to H1N1 in both young and older subjects, but the increase was greater in the young subjects (Fig. 2a). When the young and older cohorts were combined, there was a trend for a treatment effect (*p* < 0.02; Fig. 2a), but this did not remain when the cohorts were analysed separately.

#### Total antibody response to the H3N2 strain

Vaccination resulted in a significant increase in mean antibody titres to the H3N2 subunit of the vaccine (Fig. 2b). However, there was a significant age*time interaction (*p* < 0.01) reflecting the greater increase in antibody titres in response to vaccination in young subjects compared to older subjects. There was no significant effect of treatment with the synbiotic on antibody titres to H3N2 (Fig. 2b).

#### Total antibody response to the Brisbane strain

Vaccination increased mean antibody titres to the Brisbane strain (Fig. 2c). There was a significant effect of age (*p* < 0.01), with mean antibody titres to the Brisbane subunit being significantly higher in young subjects after vaccination (Fig. 2c), and there was a significant age*time interaction, reflecting the greater response to vaccination in the young subjects (Fig. 2c).

There was no overall effect of treatment when the young and older cohorts were combined, but when they were considered separately, the synbiotic was associated with a borderline effect (reduction) on the antibody response to vaccine in the young subjects (Fig. 2c).

### Effect of *B. longum* + Gl-OS on seroprotection to seasonal influenza vaccination

Baseline seroprotection to H1N1 was significantly different in the young and older subjects (Table 2). The young subjects already had a significant level of protection at baseline (GMT > 32) compared to older subjects, and this was apparent in both the placebo and the synbiotic groups (Table 2). After vaccination, seroprotection to the H1N1 strain tended to remain higher amongst the young subjects compared with the older subjects (Table 2), but there was no clear effect of treatment. There were no differences in seroprotection to H3N2 between cohorts and no effect of the synbiotic on seroprotection to H1N1 or H3N2 in either cohort (Table 2).

**Table 2:** Effect of *B. longum* + Gl-OS on seroprotection status in young and older subjects before and 4 weeks after influenza vaccination

	Seroprotection status					
Vaccine strain		Young cohort (*n* = 58)		Older cohort (*n* = 54)		*p*
		Placebo	*B. longum* + Gl-OS	Placebo	*B. longum* + Gl-OS	
		n/N (%)	n/N (%)	n/N (%)	n/N (%)	
A/California/7/2009 (H1N1)	Before	20/29 (69)^a^	13/29 (45)^a^	5/28 (18)	1/26 (4)	< 0.001
	After	29/29 (100)	27/29 (93)^a^	22/27 (81)	16/26 (62)	< 0.001
A/Perth/16/2009 (H3N2)	Before	10/29 (34)	13/29 (45)	8/28 (29)	12/26 (46)	NS
	After	26/29 (90)	27/29 (93)	25/27 (93)	22/26 (85)	NS
B/Brisbane/60/2008	Before	4/29 (14)	0/29 (0)	4/24 (17)	5/26 (19)	NS
	After	28/29 (97)	20/29 (76)	14/27 (52)	11/26 (42)	< 0.001

Data are numbers of seroprotected (n)/total (N) and % seroprotected subjects in parentheses. ‘Before’ refers to week 4 samples and ‘After’ refers to week 8 samples (ie 4 weeks after vaccination). *P* refers to results of Fisher’s exact test for differences between the cohorts with treatment groups combined; ^a^young cohort significantly different from the older cohort within same treatment group (*p* < 0.01, Fisher’s Exact test)

For the Brisbane strain, seroprotection was significantly greater in the young subjects following vaccination (Table 2), but there was no clear effect of treatment with the synbiotic.

### Effect of *B. longum* + Gl-OS on seroconversion to seasonal influenza vaccination

Seroconversion to H1N1 and H3N2 was not significantly different between the young and older subjects, although there was a trend for better seroconversion to H3N2 in the young subjects (Table 3). Young subjects demonstrated significantly greater seroconversion to the Brisbane strain than older subjects (Table 3). There was no significant effect of supplementation with the synbiotic on seroconversion to Brisbane, although it tended to reduce seroconversion in the older subjects (Table 3).

**Table 3:** Effect of *B. longum* + Gl-OS on seroconversion in young and older subjects 4 weeks after influenza vaccination (week 8)

	Seroconversion status (≥ fourfold increase in Ab titres)				
Vaccine strain	Young cohort (*n* = 58)		Older cohort (*n* = 54)		*p*
	Placebo	B. longum + Gl-OS	Placebo	B. longum + Gl-OS	
	*n*/*N* (%)	*n*/*N* (%)	*n*/*N* (%)	*n*/*N* (%)	
A/California/7/2009 (H1N1)	15/29 (52)	18/29 (62)	21/27 (78)	15/26 (58)	NS
A/Perth/16/2009 (H3N2)	20/29 (69)	22/29 (76)^a^	17/27 (63)	10/26 (38)	0.031
B/Brisbane/60/2008	23/29 (79)^a^	20/29 (69)^a^	7/27 (26)	1/26 (4)	< 0.001

Data are numbers of seroconverted (*n*)/total (*N*) and % seroconverted subjects in parentheses. *P* refers to results of Fisher’s exact test for differences between the cohorts with treatment groups combined; ^a^young cohort significantly different from the older cohort within same treatment group (*p* < 0.01, Fisher’s Exact test)

### Effect of *B. longum* + Gl-OS on levels of vaccine-specific IgA, IgG1, IgD and IgM and salivary sIgA following seasonal influenza vaccination

Levels of vaccine-specific IgA, IgG, IgD and IgM were significantly increased following influenza vaccination in both young and older subjects (Additional file 1: Figure S1). In all cases, except for IgM, increases were significantly greater in the young subjects (Additional file 1: Figure S1). There was no effect of the synbiotic on levels of vaccine-specific Ig subclasses, although there was a tendency for a smaller IgG response in the older subjects on the synbiotic relative to the placebo group (*p* = 0.03; Additional file 1: Figure S1) and an improved induction of vaccine-specific IgM and, to some degree, IgG (only at week 6) in young subjects (Additional file 1: Figure S1). There was no effect of either age or of the synbiotic on concentrations of salivary sIgA at any timepoint (data not shown).

### Effect of *B. longum* + Gl-OS on copy number of *B. longum* in faecal samples and influence of numbers of bifidobacteria on seroconversion

There was a significant increase in copy numbers of *B. longum* in faecal samples from subjects taking the synbiotic when the young and older cohorts were combined (*p* < 0.01), but although trends remained when the cohorts were analysed separately, they were not statistically significant (Additional file 2: Figure S2). Overall, no significant genus-level changes in the faecal gut microbiota, as assessed by FISH, were observed (data not shown), but seroconverters to all three subunits of the influenza vaccine tended to demonstrate a greater bifidogenic effect of the pre- and probiotic treatment compared with non-seroconverters (*p* = 0.057), suggesting that successful seroconversion may be associated with increased numbers of bifidobacteria (Additional file 3: Figure S3).

### Influence of baseline markers of immunosenescence and CMV status on intervention outcome

**Fig. 3:**
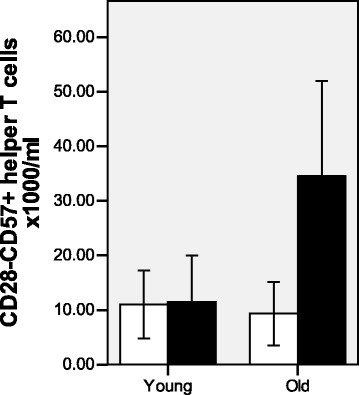
Numbers of CD28^−^CD57^+^ helper T cells at baseline differ in older subjects randomized to *B. longum* + Gl-OS and placebo. Data are absolute numbers of T-cell subsets × 1000/ml ± 2SEM for *n* = 58 young and *n* = 54 older subjects randomized to *B. longum* + Gl-OS ■ or placebo □. Data were analysed using Student’s independent *t*-tests for differences between young and older subjects. ** Denotes significant difference between treatment groups within age cohort (*p* < 0.01)

**Fig. 4:**
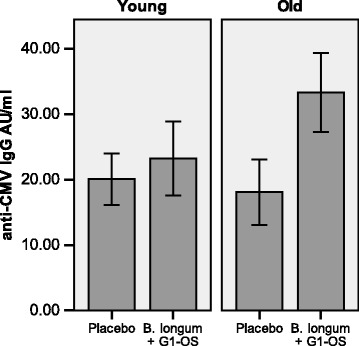
Baseline levels of anti-CMV IgG differ in older subjects randomized to *B. longum* + Gl-OS and placebo. Data are anti-CMV IgG (AU/ml) ± 2SEM for *n* = 45 young and *n* = 45 older subjects randomized to *B. longum* + Gl-OS or placebo. Data were analysed using Student’s independent t-tests for differences between young and older subjects. * Denotes significant difference between treatment groups within age cohort (*p* < 0.05)

**Fig. 5:**
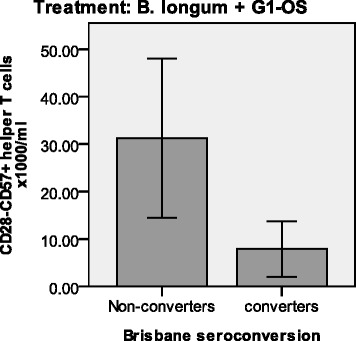
Lower numbers of circulating CD28-CD57^+^ T helper cells in seroconverters to the Brisbane subunit compared with non-converters amongst subjects randomized to *B. longum* + Gl-OS. Data are absolute numbers of T cell subset x1000/ml ± 2SE for *n* = 58 young and *n* = 54 older subjects. * Denotes significantly different from non-seroconverters (*P* < 0.05, independent values *T*-test)

Older subjects randomized to the synbiotic had a significantly higher number of senescent (CD28^−^CD57^+^) helper T cells at baseline compared with those randomized to the placebo (Fig. 3). They also had significantly higher plasma levels of anti-CMV IgG (Fig. 4) and a greater tendency for CMV seropositivity (66.7 % vs 38.1 %). CD28^−^CD57^+^ helper T cells and cytotoxic T cells were significantly higher in CMV^+^ subjects compared with age-matched CMV^−^ subjects and were positively correlated with the plasma level of anti-CMV IgG and with telomere length (data not shown). Higher numbers of these CD28^−^CD57^+^ helper T cells were associated with failure to seroconvert to Brisbane (Fig. 5), suggesting that differences in immunosenescence between the randomized groups at baseline may have influenced the outcome of the intervention.

## Discussion

This paper demonstrates marked impairment of the antibody response to influenza vaccination in older subjects. Intervention with a novel synbiotic, *B. longum* + Gl-OS failed to reverse this impairment in older subjects, and in fact there were trends for reduced seroconversion to the Brisbane subunit and a reduced IgG response. However, further immunological characterization revealed a greater degree of immunosenescence at baseline in older subjects randomized to the synbiotic, which could explain the particularly poor response of these subjects to the vaccination. This demonstrates, for the first time, that the interpretation of interventions examining the response to vaccination in older people may be highly dependent on their baseline immunological phenotype.

While there is general consensus that ageing impairs the response to influenza vaccination [13], there are very few robust studies specifically comparing responses of young and older subjects, and the most comprehensive information available is from a meta-analysis published in 2006, which concluded that clinical vaccine efficacy in older subjects was only 17–53 % compared with 70–90 % in young subjects [14]. In the current study, antibody responses to all three subunits of the influenza vaccine were impaired in the older subjects and there was significantly greater seroprotection to the H1N1 and Brisbane subunits and seroconversion to the H3N2 and Brisbane subunits in the young subjects after vaccination. Accounting for differences in baseline seroprotection between individuals and groups was a particular challenge in the current study since the B/Brisbane/60/2008 strain had previously been used in the trivalent vaccine in the 2009/2010 season and the H1N1 strain had previously been used in the monovalent swine flu vaccination during the pandemic of 2009/2010 (although only three subjects had previously been vaccinated with this). In the current study, individuals who were seroprotected at baseline were not excluded because it was considered valuable to employ a ‘real life’ scenario. Notably, the older subjects had high pre-vaccination antibody titres to the Brisbane strain and a poor seroconversion rate compared with the young subjects. On the other hand, baseline seroprotection for the H1N1 strain was greater in the young cohort, yet rates of seroconversion were similar in the two cohorts.

To date, studies which have examined the potential adjuvant properties of probiotics given in conjunction with influenza vaccines in healthy adults have shown promise in their ability to beneficially modulate the humoral response to influenza, but robust data is still lacking [6, 15]. The effects of probiotics on the response to influenza vaccination in older adults is of particular importance, given the consequences of respiratory infections in older people [16]. Among free-living Chilean subjects over 70 years old, a complete nutritional formula containing a range of nutrients and vitamins plus the probiotic *Lactobacillus paracasei* (NCC 2461) and the prebiotic fructo-oligosaccharide was associated with a significantly lower incidence of respiratory infection, but had no effect on antibody responses to vaccination [17]. Provision of a probiotic drink containing *Lactobacillus paracasei* ssp. *paracasei* (Actimel®) to healthy elderly volunteers (> 70 years) resulted in higher influenza virus-specific antibody titres post-vaccination, although these differences were only statistically significant within the confirmatory phase of this study [18]. A small study of 27 elderly (mean age > 85) subjects provided with *Bifidobacterium longum* BB536, reported significantly lower incidence of influenza and fever in the probiotic group [19]. However, the largest study to date investigated the effect of *Lactobacillus casei* Shirota (*Lc*S) on the susceptibility to respiratory symptoms and influenza vaccination responses among 737 nursing home residents (mean age > 80 year) and reported no significant differences in antibody titres, seroconversion or seroprotection 4 and 22 weeks after vaccination, and no effect on reported respiratory symptoms [20]. Thus the evidence is not clear cut and it is possible that there are differences in the immunomodulatory potential of different strains.

In the current study, the response of the young and older subjects to the intervention differed to some degree. In older subjects consuming the synbiotic, there was a trend for reduced seroconversion to the Brisbane subunit, whereas in the young subjects, there were trends for enhanced production of vaccine-specific IgM and, to some extent, IgG. Increased production of vaccine-specific IgM and IgG following intervention with probiotics has been reported in several other studies [21–25]. The possibility that there is a differential immune response to probiotics in young vs older subjects has also been explored to some extent. You et al. [12] demonstrated that peripheral blood mononuclear cells (PBMC) from older subjects (60–85y) were more responsive to the immunoregulatory effects (IL-10 induction) of two strains of bifidobacteria than young subjects (18–30y), whereas PBMC from young subjects were more responsive to the immunostimulatory effects (IL-12 induction) of two strains of lactobacilli. Further studies demonstrated that probiotics (including the strain employed in the current study) increased the responsiveness of DCs in older subjects to a greater degree than young subjects, but this was not sufficient to overcome the impact of immunosenescence in a mixed leukocyte reaction [26]. The choice of probiotic, particularly for older individuals, is a matter of debate and it has been suggested that ‘successfully aged’ donors of probiotic strains might survive better in an older host and achieve a more suitable equilibrium with the resident microbiota [9]. *Bifidobacterium longum bv. infantis* CCUG 52,486 is an example of a strain present in particularly healthy subjects aged > 90y, which has subsequently been demonstrated to have particular ecological fitness and anti-pathogenic effects in vitro [27] and immunomodulatory effects which are strongly influenced by the age of the host [12, 26]. The effects of prebiotics on immune function are less well characterized than those of probiotics, but are suggested to be mainly indirect via modulation of the gut microbiota. In the current study, the prebiotic Gl-OS was used chiefly to support the colonization of the probiotic, and although an independent effect of the prebiotic on the gut microbiota cannot be excluded, the fact that the intervention increased copy numbers of *B. longum* while having no significant effect on the fecal microbiota at genus level suggests that the prebiotic was selectively supporting the growth of the probiotic strain *in vivo*.

The results of the intervention must take into account the potential influence of baseline differences in immunosenescence, which in the current study were revealed after the study was completed and occurred entirely by chance. T cells are particularly susceptible to senescence, resulting in loss of CD28; repeated antigenic exposure, for example to cytomegalovirus (CMV), is suggested to play a major role in this [28, 29]. Latent infection with CMV has been demonstrated to result in a poor response to infection and vaccination [29]. In the current study, not only did older subjects randomized to the synbiotic have a significantly higher number of senescent (CD28^−^CD57^+^) helper T cells at baseline compared with those randomized to the placebo, they also had significantly higher plasma levels of anti-CMV IgG and a greater tendency for CMV seropositivity. Moreover, higher numbers of CD28^−^CD57^+^ helper T cells were associated with failure to seroconvert to Brisbane, strongly suggesting that the subjects randomized to the synbiotic were already at a significant disadvantage in terms of likely ability to respond to the vaccine compared with those randomized to the placebo and that differences in immunosenescence between the randomized groups at baseline may have influenced the outcome of the intervention. Future work therefore needs to consider prospective randomization of subjects based on robust immunological markers; this is challenging given the wide range of potential markers and uncertainty regarding their predictive value.

In this paper, we report the effects of the synbiotic on the humoral arm of the immune response only. However, recognizing the importance of the T cell response following vaccination, and the potential effects of the synbiotic on adaptive immunity [30], we conducted extensive analysis of antigen-specific T cell activation following in vitro vaccine recall challenge. We observed that while the synbiotic increased the responsiveness of cytotoxic T cells to re-stimulation with the vaccine in young subjects, it decreased responsiveness in the older subjects (unpublished data). Thus, the impact of greater immunosenescence in the older subjects randomized to the synbiotic group appears to be significant, extending beyond the humoral arm of the immune response and influencing the T cell response to the vaccine.

## Conclusion

In conclusion, we demonstrate marked impairment of the antibody response to influenza vaccination in older subjects and failure of the synbiotic to reverse this impairment. However, the older subjects randomized to the synbiotic were revealed to be at a significant disadvantage due to significantly higher numbers of senescent (CD28^−^CD57^+^) helper T cells at baseline compared with those randomized to the placebo, as well as significantly higher plasma levels of anti-CMV IgG and a greater tendency for CMV seropositivity. Thus, baseline differences in immunosenescence between the randomized groups are likely to have influenced the outcome of the intervention, highlighting the need for detailed immunological characterization of subjects prior to interventions.

## Methods

### Ethics and trial registration

The study protocol was reviewed and approved by the University of Reading Research Ethics Committee (project number: 10/09) the National Health Service (NHS) Research Ethics Committee for Wales (10/MRE09/5). The trial was registered with clinicaltrials.gov (Identifier: NCT01066377) and conducted according to the guidelines laid down in the Declaration of Helsinki.

### Participants

Prior to the influenza season of 2010–2011, young (18–35 y) and older (60–85 y) healthy adults were recruited from the population in and around Reading (UK) through newspaper and poster advertisements, email and radio. Inclusion criteria were: a signed consent form, age 18–35 y or 60–85 y, body mass index (BMI) 18.5–30 kg/m^2^, good general health, as determined by medical questionnaires and laboratory data from screening blood and urine sample (fasting glucose, erythrocyte sedimentation rate, full blood count, liver function tests, renal profile, dipstick urinalysis), not pregnant, lactating or planning a pregnancy. Exclusion criteria included: allergy to the influenza vaccine, HIV infection, diabetes requiring any medication, asplenia and other acquired or congenital immunodeficiences, any autoimmune disease, including connective tissue diseases, malignancy, cirrhosis, connective tissue diseases, current use of immunomodulating medication (including oral and inhaled steroids), self–reported symptoms of acute or recent infection (including use of antibiotics within last 3 months), taking lactulose or any other treatment for constipation, alcoholism and drug misuse. Additional exclusion criteria for older volunteers included: laboratory data which were outside the normal range for this age group AND outside the ranges specified in the SENIEUR protocol [31], Barthel Index score of < 16/100, cumulative illness rating scale (CIRS) score of > 15 [32]. Additional exclusion criteria for the young subjects included laboratory data which were outside the normal range and influenza vaccination in the previous 12 months.

### Sample size

The primary outcome of the trial was the antibody response to vaccination, incorporating mean antibody titres, vaccine-specific Ig subclasses and seroprotection and seroconversion. Power calculations were based on mean antibody titres. Since the influenza vaccine is trivalent, it is unlikely that an intervention will alter the response to all three subunits in the same way. For example, in the study of Davidson et al. [33], there was no effect of probiotic on mean antibody titres in response to the H1N1 subunit, whereas the responses to both H3N2 and the B subunit were improved (72 vs 51 [SD 16.5] for H3N2 and 31 vs 25 [SD 7.1] for B subunit). Based on the smaller effect size for the B subunit, a sample size of 26 subjects per group within each cohort was determined to be sufficient for a two-tailed significance level of 5 % and a power of 80 %; this was adjusted to 30 subjects per group to allow for dropouts. Data on the co-primary endpoints, immunoglobulin subclasses, seroprotection and seroconversion, is very sparse, but a sample size of 26 subjects per group within each cohort was determined to be sufficient for a 376 mg/dL difference in circulating IgG levels in response to influenza vaccination, with an SD of 438 mg/dL, a two-tailed significance level of 5 % and a power of 80 % [23]. A total of 62 young subjects and 63 older subjects entered the study and 58 young and 54 older subjects completed the study (Fig. 1). Two subjects experienced adverse effects (gastrointestinal bloating) during the study, one on the placebo group and one in the *B. longum* + Gl-OS group; both withdrew from the study.

### Study design

**Fig. 6:**
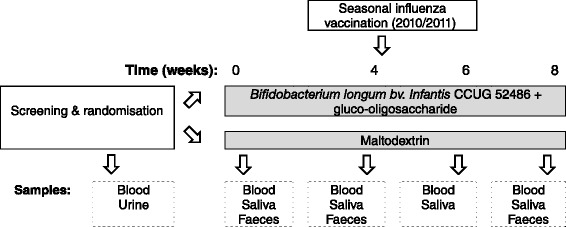
Study design

Subjects consumed *Bifidobacterium longum bv. infantis* CCUG 52,486 (*B. longum*, 10^9^ CFU in 1 g skim milk powder/day) combined with gluco-oligosaccharide (Gl-OS (BioEcolians, Solabia); 8 g/day) in a double-blind, placebo controlled randomised parallel study design for 8 weeks. The synbiotic approach was selected because in vitro data examining the growth and survival of this strain indicated that it was very vulnerable compared with other strains, but survived much better in the presence of an oligosaccharide substrate (data not shown). When comparing a number of possible substrates, the low water activity of Gl-OS, combined with its ability to support the growth of the probiotic strain, made it a clear choice for a powdered product. This prebiotic also has bifidogenic effects in batch culture models [34]. The placebo used was maltodextrin (9 g/day); both the placebo and the pre- and probiotic were sourced, packaged and blinded by BioAgro S.A. (Italy). The powders were consumed sprinkled into water or milk or with breakfast cereal. Microbiological safety of the product was independently verified by Leatherhead Food Research associates (UK) prior to commencement of the study and viability of the probiotic strain was confirmed on random samples on a weekly basis during the study. During the 3 weeks prior to the study and during the intervention itself, subjects were requested not to consume fermented products such as yogurts, kefir etc. or pre- or probiotic products. Dietary records were used to confirm avoidance of such foods. Subjects were randomized by a research nurse not involved in the analysis according to gender, age and BMI to receive the probiotic or placebo by covariate adaptive randomization [35]. All investigators were blinded to the treatments. After 4 weeks, subjects were administered with a single dose of the influenza vaccine (Influvac®sub-unit 2010/2011 season, Abbott Biologicals B.V., lot number 1070166) containing A/California/7/2009 (H1N1), A/Perth/16/2009 (H3N2) and the B/Brisbane/60/2008- like strain by intra-muscular injection in the deltoid. Vaccination was carried out by a research nurse in the presence of a qualified clinician (MG). Details of the study schedule and samples collected are detailed in Fig. 6. Compliance was assessed by counting returned sachets and by copy numbers of *B. longum*, assessed by qPCR. None of the subjects in the young cohort had previously received seasonal influenza vaccination or swine flu vaccination. Three subjects in the older cohort had received swine flu vaccination, and forty subjects had previously been vaccinated for seasonal influenza, of whom 37 had been vaccinated in the 2009/2010 period.

### Blood sample processing

For serum, blood was collected into serum separator tubes and left at room temperature for 30mins to allow coagulation. Samples were centrifuged at 1300 × g for 10 min and aliquots of serum were collected and stored at −80 °C prior to analysis.

### Hemagglutination inhibition assay

Serum samples (in duplicate) were sent to the Health Protection Agency (London, UK) for analysis of total antibody responses to each of the 3 viral subunits. This technique is based on the ability of specific anti-influenza antibodies to inhibit hemagglutination of red blood cells by influenza virus hemagglutinin protein. Serum antibody titers measured by hemagglutination inhibition (HI) assay was performed using an 8-step dilution protocol (1:8 to 1:1024). Serum samples were titrated in duplicate, and samples showing titres of 1024 were further titrated up to 1:4096. Titres which were < 8 (below the detection limit) were expressed as 4, to represent half of the detection threshold. Post-vaccination antibody titres were not normally distributed, and were therefore log transformed.

### Analysis of vaccine-specific Ig subclasses

Vaccine-specific Ig subclasses were analysed using an in-house ELISA method. Briefly, 96-well maxisorb ELISA plates (Serotec) were coated with 500 ng/ml of the vaccine (Influvac sub-unit 2010/2011) in coating buffer (0.5 M Na_2_CO_3_). After overnight incubation at 4 °C, plates were washed three times with wash solution (50 nM TRIS, 0.14 M NaCl, 1 % BSA, 0.2 % Tween-20 in dH_2_0). Blocking buffer (5 % BSA in PBS) was added and plates were incubated for 1 h at 37 °C. After three washes, plasma samples (neat for IgA, IgD, IgM, and diluted 1:100 in PBS for IgG1) were added, and plates were incubated for a further hour at RT. Plates were then washed to remove non-specific binding antibodies and mouse anti-human IgA, IgG, IgM or IgD was added. IgA, IgG1, and IgM were diluted with PBS 1:1000, whereas IgD was diluted 1:500. After a 1 h incubation, diluted horse radish peroxidase conjugated goat anti-mouse IgG (H/L) (AbD Serotec, Oxfordshire, UK) was added and the plate was incubated for a further 1 h at RT. Substrate (3.3’, 5.5’ tetramethylbenzidine) was added and the reaction was stopped with 0.16 M sulphuric acid. Plates were read at 450 nm in a micro-plate autoreader (Thermo Scientific) and the results were presented as a mean optical density units (OD) ± SEM.

To minimize inter-assay variation, three quality control samples were included in every assay, consisting of post-vaccination plasma samples from two young and one older subject.

### Analysis of salivary sIgA

Saliva samples were collected by passive drooling into a sterile screw-cap tube, centrifuged at 13,000 × g for 10 min at 4 °C to remove cells and debris and stored at −80 °C until analysis, which was completed within 6 months. The concentration of sIgA in saliva was analysed by sandwich ELISA using a commercially available kit (Immunodiagnostik AG, Bensheim, Germany) in accordance with the manufacturer’s instructions.

### Analysis of anti-CMV IgG antibodies

Concentrations of anti-CMV IgG antibodies were analysed by ELISA according to the manufacturer’s instructions (ab108724 Anti-Cytomegalovirus (CMV) IgG Human Elisa Kit, Abcam, UK) and read in a microplate reader (GENios) at 450 nm, with 620 nm as a reference wavelength.

### T cell phenotyping

Peripheral blood mononuclear cells (PBMC: 1 × 10^6^) were stained with the following fluorochrome-conjugated monoclonal antibodies: (PerCP) labelled anti-CD3, (AmCyan) labelled anti-CD4, (APC-Cy7) labelled anti-CD8, (PE-Cy7) labelled anti-CD25, (Pacifice Blue) labelled anti-CD28, (APC) labelled anti-CD57, (FITC) labelled anti-CD26, (PE) labelled anti-CD127 in TruCOUNT tubes (BD Biosciences), washed and fixed before analysis by multiparamter flow cytometry (FACS Canto II, BD Biosciences) using BD *FACSDiva*™ software.

### Relative telomere length

Telomere length of PBMC was measured by using the DAKO Telomere PNA Kit/FITC for Flow Cytometry (DAKO, UK), according to the manufacturer’s instructions. Relative telomere length (RTL) was determined by comparing isolated PBMC with a control cell line (1301; subline of the Epstein–Barr virus (EBV) genome negative T-cell leukaemia line CCRF-CEM; Sigma-Aldrich Company Ltd, UK). Quantification of RTL was carried out using an Accuri™ C6 flow cytometer (BD Biosciences, Oxford, UK).

### Quantification of faecal *Bifidobacterium longum*

Freshly voided faecal samples were collected in sterile plastic pots and stored at −20 °C.

DNA was extracted from faecal samples using a commercial kit (Fast DNA Spin kit, MP Biomedicals, Cambridge, UK) according to the manufacturers’ instructions. Quantity and purity of the DNA was assessed using NanoDrop (Thermo Fisher Scientific Inc., Loughborough, UK). Real-time quantitative PCR was performed in triplicate for enumeration of *Bifidobacterium longum* with the Rotor-Gene 6000 (Corbett, Mortlake, Australia) using 5 μL of extracted faecal DNA in 20 μL PCR master-mix according to Haarman and Knol (2005). The reaction was carried out after 1 cycle at 95 °C for 10 min, followed by 45 cycles at 95 °C × 10, 62 C° per 60 s, 72 °C per 40 s. Standard quantification was carried out using serially diluted DNA extract of *Bifidobacterium longum bv. infantis* CCUG 52,486 strain enumerated by plate count.

### Fluorescence in situ hybridisation

Freshly voided faecal samples were collected in sterile plastic pots, diluted 1 in 10 (wt:wt) in PBS and homogenised in a Stomacher 400 (Seward, Norfolk, United Kingdom) for 2 min. A 15 ml sample of faecal slurry was vortexed with 2 g of 3 mm diameter glass beads and then centrifuged to remove particulate matter (1500 × g, 2 min). Supernatant was fixed in paraformaldehyde [1:4 v/v in 4 % PFA in 0.1 mol PBS, pH 7.2] for 4 h at 4 °C, centrifuged (13,000 g 5 mins), washed twice with 0.1 M PBS, resuspended in 1:1 PBS:ethanol and stored at −20 °C. Oligonucleotide probes used were Cy-3 labeled and synthesized by Sigma-Aldrich (Poole, UK). Probes used were Bif164, Bac303, Chis150, Lab158, ATO291, Enter1432, Fprau655 and EUB338I/II/III specific for *Bifidobacterium* spp.*, Bacteroides/Prevotella* group*, Clostridium* clusters I and II (including *C. perfringens* and *C. histoliticum*)*, Lactobacillus/Enterococcus* subgroup, *Atopobium, Enterobacterium* group, *Faecalibacterium* cluster and total bacteria respectively. Samples were hybridized as described [36]. Data is expressed as log10 counts per g dry weight faeces.

### Self-reported illness

Subjects in the older cohort who received the influenza vaccination during October/November 2010 in accordance with UK NHS vaccination schedules (*n* = 43) were requested to record incidence of any cold or flu like symptoms in the 6 months post-vaccination. However, the numbers were too small to be statistically meaningful and are therefore not included.

### Statistical analysis

Data were analysed using SPSS software (version 21). For the primary and continuous secondary endpoints, a Linear Mixed Model (LMM) was implemented. A first order autoregressive covariance structure was selected AR (1), with fixed factors of time (repeated measures), age and treatment and subject as a random effect. The main effects are reported first, followed by all two-way interactions of the variables. Following this initial analysis, the data were split by cohort (young/older) and the analysis was repeated in the same manner to determine time and treatment effects within each cohort. Vaccine-specific immunoglobulin subclass data were analysed as change following vaccination. The distribution of the data was checked using the Kolmogorov-Smirnov test. If data were not normally distributed, they were log transformed. Antibody titre data were transformed with binary log (log_2_*n*) and were not analysed as change from baseline. Seroprotection, seroconversion and self-reported illness data were analysed by the Fisher’s exact test. To account for multiple primary endpoints, two sided *P* values of 0.01 or less were considered statistically significant. All missing data were classed as missing at random and only available data were analysed.

## Additional files

Additional file 1: Figure S1. Effect of *B. longum* + Gl-OS on levels of vaccine-specific IgA, IgG1, IgD and IgM. Data are optical density units (OD_450nm_) ± 2 SEM, change from baseline (week 4) for *n* = 54–58 subjects per group, 2 (week 6) and 4 (week 8) weeks after vaccination. ☐ Maltodextrin, (gray square) *B. longum* + Gl-OS. Data were analysed using a Linear Mixed Model (LMM) with fixed factors of time, age and treatment. For IgA (plot A), there was a significant effect of age (*p* < 0.001) and time (*p* < 0.001) and a significant age*time interaction (*p* < 0.001). Data split by cohort showed a significant effect of time in both the young (*p <* 0.01) and older (*p* < 0.001) cohorts. For IgG1 (plot B), there were significant effects of time (*p* < 0.001) and age (*p* < 0.001). Data split by cohort showed a significant effect of time (*p* < 0.001) and a trend for a treatment effect (*p* = 0.03) in the older cohort, and a significant effect of time (*p* < 0.001) in the young cohort. For IgD (plot C), there were significant effects of age (*p* < 0.001) and time (*p* < 0.001) for the combined cohorts and a significant effect of time in both the young (*p* < 0.001) and older cohorts (*p* < 0.001) when considered separately. For IgM (plot D), there was a significant effect of time (*p* < 0.001), but no effect of age or treatment. (DOCX 52 kb) Additional file 2: Figure S2. Copy numbers of *B. longum* + Gl-OS in faecal samples. Data are mean ± 2SEM for *n* = 58 young and *n* = 54 older subjects. ☐ Maltodextrin, (gray square) *B. longum* + Gl-OS. Data were analysed using a Linear Mixed Model (LMM) with fixed factors of time, age and treatment. There were no statistically significant effects, either for combined or separate cohorts. (DOCX 25 kb) Additional file 3: Figure S3. Effect of *B. longum* + Gl-OS on numbers of bifidobacteria in seroconverters vs non converters. Data are mean ± 2SEM for *n* = 18 seroconverters (to all three subunits) and *n* = 135 non-converters in the placebo group and *n* = 14 seroconverters and *n* = 36 nonconverters in the *B. longum* + GlOS group. Trend for greater increase in bifidobacteria in seroconverters compared with non-converters in the *B. longum* + GlOS group (*p* = 0.057). (DOCX 26 kb)

## Abbreviations

BSA: bovine serum albumin
CIRS: cumulative illness rating scale
ELISA: enzyme-linked immunosorbent assay
Gl-OS: gluco-oligosaccharide
Ig: immunoglobulin
PBMC: peripheral blood mononuclear cells
PBS: phosphate-buffered saline
RPMI: Roswell Park Memorial Institute
URTI: upper respiratory tract infection.
