# Supplementary material for: Effect of a synbiotic on the response to seasonal influenza vaccination is strongly influenced by degree of immunosenescence
Source: Immun Ageing. 2016 Mar 15;13:6. doi: 10.1186/s12979-016-0061-4 (PMC4793545; doi:10.1186/s12979-016-0061-4)
Supplement: Additional file 1: Figure S1. — Effect of B. longum + Gl-OS on levels of vaccine-specific IgA, IgG1, IgD and IgM. Data are optical density units (OD450nm) ± 2 SEM, change from baseline (week 4) for n = 54–58 subjects per group, 2 (week 6) and 4 (week 8) weeks after vaccination. ☐ Maltodextrin, (gray square) B. longum + Gl-OS. Data were analysed using a Linear Mixed Model (LMM) with fixed factors of time, age and treatment. For IgA (plot A), there was a significant effect of age (p < 0.001) and time (p < 0.001) and a significant age*time interaction (p < 0.001). Data split by cohort showed a significant effect of time in both the young (p < 0.01) and older (p < 0.001) cohorts. For IgG1 (plot B), there were significant effects of time (p < 0.001) and age (p < 0.001). Data split by cohort showed a significant effect of time (p < 0.001) and a trend for a treatment effect (p = 0.03) in the older cohort, and a significant effect of time (p < 0.001) in the young cohort. For IgD (plot C), there were significant effects of age (p < 0.001) and time (p < 0.001) for the combined cohorts and a significant effect of time in both the young (p < 0.001) and older cohorts (p < 0.001) when considered separately. For IgM (plot D), there was a significant effect of time (p < 0.001), but no effect of age or treatment. (DOCX 52 kb) [file 12979_2016_61_MOESM1_ESM.docx]

Supplementary Figure 1

A


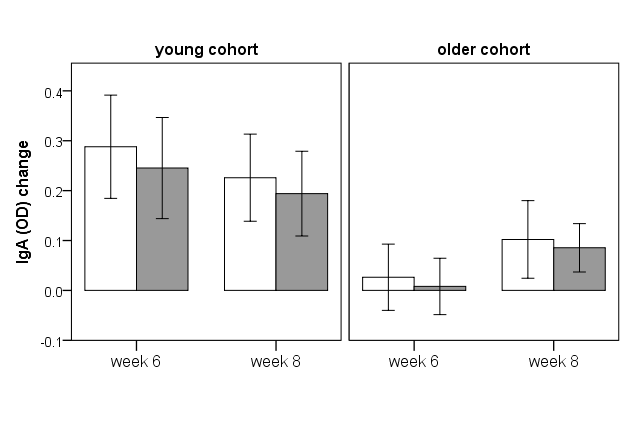


B


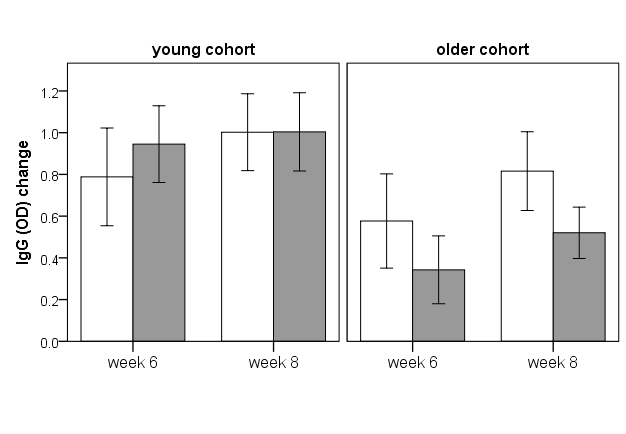


C


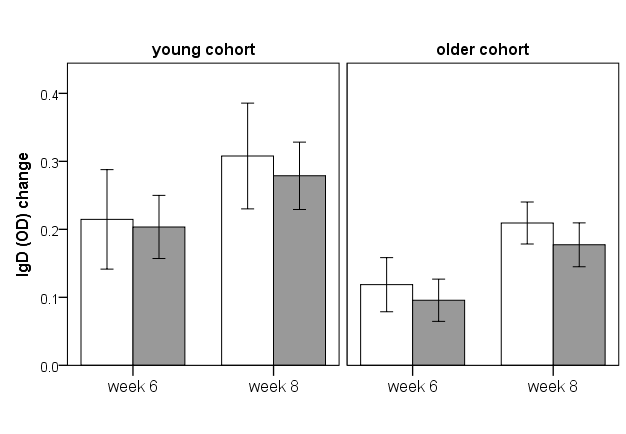


D


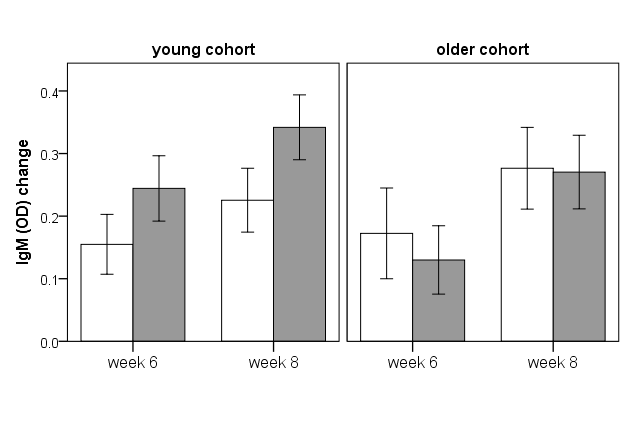


Supplementary Figure 1. Effect of *B. longum* + Gl-OS on levels of vaccine-specific IgA, IgG1, IgD and IgM. Data are optical density units (OD_450nm_) ± 2 SEM, change from baseline (week 4) for n= 54-58 subjects per group, 2 (week 6) and 4 (week 8) weeks after vaccination. ☐ Maltodextrin, ◼ *B. longum* + Gl-OS. Data were analysed using a Linear Mixed Model (LMM) with fixed factors of time, age and treatment. For IgA (plot A), there was a significant effect of age (*p*<0.001) and time (*p*<0.001) and a significant age*time interaction (*p*<0.001). Data split by cohort showed a significant effect of time in both the young (*p<*0.01) and older (*p*<0.001) cohorts. For IgG1 (plot B), there were significant effects of time (*p*<0.001) and age (*p*<0.001). Data split by cohort showed a significant effect of time (*p*<0.001) and a trend for a treatment effect (*p*=0.03) in the older cohort, and a significant effect of time (*p*<0.001) in the young cohort. For IgD (plot C), there were significant effects of age (*p*<0.001) and time (*p*<0.001) for the combined cohorts and a significant effect of time in both the young (*p*<0.001) and older cohorts (*p*<0.001) when considered separately. For IgM (plot D), there was a significant effect of time (*p*<0.001), but no effect of age or treatment.
