# Supplementary material for: Effect of a synbiotic on the response to seasonal influenza vaccination is strongly influenced by degree of immunosenescence
Source: Immun Ageing. 2016 Mar 15;13:6. doi: 10.1186/s12979-016-0061-4 (PMC4793545; doi:10.1186/s12979-016-0061-4)
Supplement: Additional file 2: Figure S2. — Copy numbers of B. longum + Gl-OS in faecal samples. Data are mean ± 2SEM for n = 58 young and n = 54 older subjects. ☐ Maltodextrin, (gray square) B. longum + Gl-OS. Data were analysed using a Linear Mixed Model (LMM) with fixed factors of time, age and treatment. There were no statistically significant effects, either for combined or separate cohorts. (DOCX 25 kb) [file 12979_2016_61_MOESM2_ESM.docx]

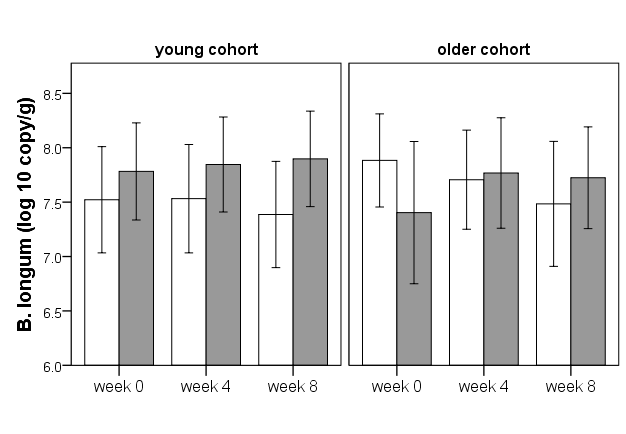


**Supplementary Figure 2. Copy numbers of *B. longum* + Gl-OS in faecal samples**

Data are mean ± 2SEM for n=58 young and n=54 older subjects. ☐ Maltodextrin,  *B. longum* + Gl-OS. Data were analysed using a Linear Mixed Model (LMM) with fixed factors of time, age and treatment. There were no statistically significant effects, either for combined or separate cohorts.
