# Supplementary material for: Effect of a synbiotic on the response to seasonal influenza vaccination is strongly influenced by degree of immunosenescence
Source: Immun Ageing. 2016 Mar 15;13:6. doi: 10.1186/s12979-016-0061-4 (PMC4793545; doi:10.1186/s12979-016-0061-4)
Supplement: Additional file 3: Figure S3. — Effect of B. longum + Gl-OS on numbers of bifidobacteria in seroconverters vs non converters. Data are mean ± 2SEM for n = 18 seroconverters (to all three subunits) and n = 135 non-converters in the placebo group and n = 14 seroconverters and n = 36 nonconverters in the B. longum + GlOS group. Trend for greater increase in bifidobacteria in seroconverters compared with non-converters in the B. longum + GlOS group (p = 0.057). (DOCX 26 kb) [file 12979_2016_61_MOESM3_ESM.docx]

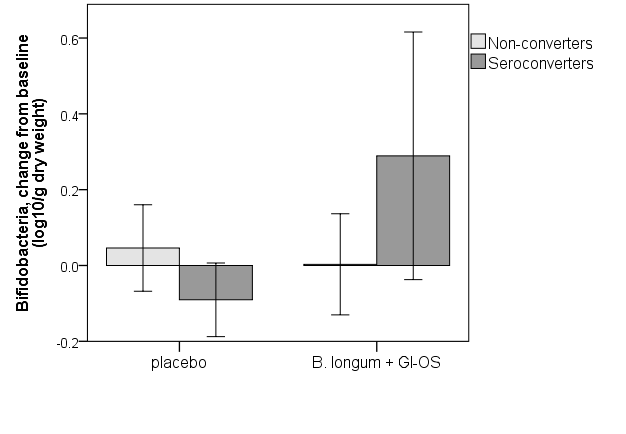


**Supplementary Figure 3. Effect of *B. longum* + Gl-OS on numbers of bifidobacteria in seroconverters vs non converters**

Data are mean ± 2SEM for n=18 seroconverters (to all three subunits) and n=135 non-converters in the placebo group and n=14 seroconverters and n=36 nonconverters in the *B. longum* + GlOS group. Trend for greater increase in bifidobacteria in seroconverters compared with non-converters in the *B. longum* + GlOS group (*p*=0.057).
